# Breeding system and pollination of two closely related bamboo species

**DOI:** 10.1093/aobpla/plx021

**Published:** 2017-05-29

**Authors:** Ling-Na Chen, Yong-Zhong Cui, Khoon-Meng Wong, De-Zhu Li, Han-Qi Yang

**Affiliations:** 1 Research Institute of Insect Resources, Chinese Academy of Forestry, Bailongsi, Panlong District, Kunming 650233, China; 2 Singapore Botanic Gardens, 1 Cluny Road, Singapore 259569, Singapore; 3 Key Laboratory of Biodiversity and Biogeography, Kunming Institute of Botany, Chinese Academy of Sciences, No. 132, Lanhei Road, Panlong District, Kunming 650201, China

**Keywords:** Breeding system, *Dendrocalamus membranaceus*, *Dendrocalamus sinicus*, floral morphology, pollination limitation, pollinators, woody bamboo

## Abstract

An understanding of the breeding systems and pollination of agriculturally important plants is critical to germplasm improvement. Breeding system characteristics greatly influence the amount and spatial distribution of genetic variation within and amongst populations and influence the rarity and extinction vulnerability of plant species. Many woody bamboos have a long vegetative period (20–150 years) followed by gregarious monocarpy. Relatively, little is known about their pollination and breeding systems. We studied these characteristics in wild *Dendrocalamus membranaceus* populations and cultivated *Dendrocalamus sinicus* populations distributed in the Yunnan Province of China. Floral morphology, flower visitors and breeding system were studied from 2013 to 2015. Both bamboos were protogynous, but flowering periods of florets overlapped providing opportunities for self-pollination amongst florets, especially in *D. membranaceus*. There was no agamospermy in either species. Seed set of *D. sinicus* was low (0.42 ± 0.42 %) under natural pollination but higher (8.89 ± 2.55 %) after artificial xenogamy. Seed set of *D. membranaceus* was higher (7.49 ± 0.82 %) in mass flowering populations and 2.14 ± 0.25 % in sporadically flowering populations. The Asian honeybee *Apis cerana* could provide cross-pollination of *D. membranaceus* and *D. sinicus*, and flower visitation peaked at 1000–1200 h. Pollination limitation due to lack of pollinators or pollen was detected in the cultivated populations of *D. sinicus* and sporadically flowering populations of *D. membranaceus*. Pollination limitation was not obvious within mass flowering populations. Hand pollination could significantly increase seed set of these two bamboo species. *Dendrocalamus membranaceus* and *D. sinicus* were self-compatible and have a mixed-mating system with outcrossing being pre-dominant. Their seed production was limited by the quantity of pollen and pollinator activity. Honeybees were observed as effective pollinators.

## Teaser

Breeding system characteristics of woody bamboo species are still little known although this knowledge is important for research on genetic improvement and conservation of bamboo forests. The present study is the first report on variation in breeding system from mass and sporadically flowering bamboo populations. The results demonstrate that two Dendrocalamus species have a mixed mating system with self-compatibility, predominant outcrossing, and no agamospermy. Pollen limitation and scarcity of wild pollinators influence sexual reproduction in sporadically flowering populations of both species.

## Introduction

The breeding system is a key component of plant sexual reproduction. Many studies have suggested that the health and maintenance of plant populations is strongly affected by breeding characteristics, such as flower phenology, self-compatibility and the breeding system (Nair *et al.* 2004; Gan *et al.* 2013). Understanding the reproductive biology of agriculturally important crops is crucial for developing genetic improvement strategies and establishing appropriate conservation measures (Nair *et al.* 2004; Rodríguez-Pérez 2005).

Bamboos (Poaceae: Bambusoideae) include ca. 88–115 genera and >1400 species. They are widely distributed, especially in the tropical and subtropical regions of the world, but absent in Europe and Antarctica (Li *et al.* 2006; Bamboo Phylogeny Group 2012). Bamboo species provide food and raw materials for construction, paper pulp and manufacturing. On the basis of the presence or absence of strongly lignified culms, bamboos are divided into two types: woody and herbaceous (Wysocki *et al.* 2015). Many woody bamboos have an unusual life cycle, characterized by a very long vegetative period of several decades to over 150 years followed by gregarious monocarpy (Budke *et al.* 2010). In some woody bamboos, such as *Ochlandra travancorica*, *Sasa kurilensis*, *Chusquea ramosissima* (Venkatesh 1984; Makita 1992; Montti *et al*. 2011), several different types of flowering occur. These include mass flowering (the flowering of most clumps in a population over a wide range) and sporadic flowering (the flowering on a small scale or the flowering of a minority of clumps in a population). Flowering studies have mainly focused on bamboo clump dieback and the ecological impact after flowering, especially in the recruitment of associated overstory tree species following mass flowering of bamboos (Campanello *et al.* 2007; Baskin 2009; Giordano *et al.* 2009). Fewer studies have focused on the natural regeneration of woody bamboos after flowering (Xie *et al.* 2016). However, due to such unpredictable flowering episodes, little is known about the breeding system and pollination of woody bamboos; such knowledge would be useful for their genetic improvement, such as better adaptation to a broader range of growth conditions (Kittelson and Maron 2000; Mousset *et al.* 2016).

As in other grasses, wind pollination is common in bamboos (Janzen 1976; Nadgauda *et al.* 1993). Insect visits to bamboo flowers have been recorded frequently although the effectiveness of insects as bamboo pollinators is controversial (Janzen 1976; Venkatesh 1984; Nadgauda *et al.* 1993; Huang *et al.* 2002). However, pollination is often dependent on mutualistic interactions with visiting insects (Gan *et al.* 2013). Huang *et al.* (2002) have provided the only study documenting honeybee-assisted pollination in addition to wind pollination in bamboos. Other studies have demonstrated bamboos being dichogamous and protogynous (Venkatesh 1984; Nadgauda *et al.* 1993; Jijeesh *et al.* 2012). Unfortunately, there is no direct information as to whether bamboos are obligate outcrossers.

**Figure 1.**
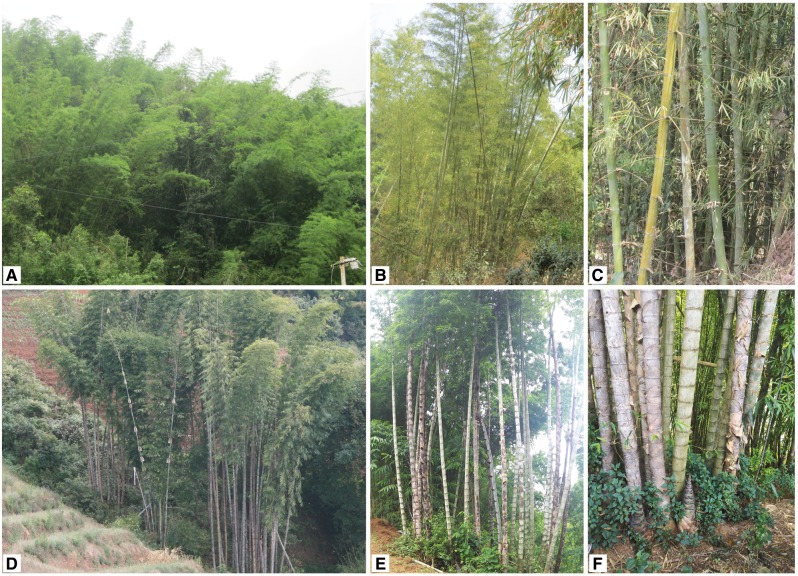
Vegetative form of *D. membranaceus* (A‒C) and *D. sinicus* (D‒F).

*Dendrocalamus* is a genus of woody bamboos with ∼52 species (Ohrnberger 1999). The species are widely distributed in the tropical and subtropical regions of Asia (Nguyen and Xia 2013). Other than reports of flowering and floral descriptions, a detailed account of reproductive biology is lacking for any of these species. *Dendrocalamus membranaceus* and *Dendrocalamus sinicus* are large woody sympodial bamboos (Fig. 1). They are economically important as vegetable crops and for providing raw materials for furniture, construction and industrial paper pulp (Li *et al.* 2006). *Dendrocalamusmembranaceus* grows in Myanmar, Laos, north Thailand, northern Vietnam and China at elevations of 500–1000 m, and is common in the Lancang-Mekong River valley (Xie *et al.* 2016). It is an important vegetation cover for soil and water conservation in parts of southwest China (Xie *et al.* 2016). *Dendrocalamussinicus* is known mainly in cultivation and is endemic to Yunnan, China, in the mountainous areas of southwestern Yunnan at 500–1800 m. It has been proposed as a protected species because of its limited distribution, rarity and continuous reduction of germplasm, as well as low seed set (Yang *et al.* 2010; Gu *et al.* 2012). These two *Dendrocalamus* species represent a common wild species and a rare, but commonly cultivated, species (Fig. 1; Yang *et al.* 2010; Xie *et al.* 2016). Since 2008, the species have started to flower sporadically or gregariously following prolonged severe drought in Yunnan, providing an opportunity to study their reproductive biology (Xie *et al.* 2016).

We examined the breeding system and pollination of *D. membranaceus* and *D. sinicus*, by studying floral characters, flower visitors, and the mating systems found in mass and sporadically flowering populations. Our goals were to determine the breeding system of two woody bamboos and document aspects of the reproductive biology that may be useful for research on genetic improvement and conservation of *Dendrocalamus* bamboo forests.

## Methods

### Species characteristics and occurrence

*Dendrocalamus* *membranaceus* individuals reach heights of 10–20 m and diameters of 7–15 cm. Clumps consist of culms sprouted from the lateral buds of shortened rhizome. Each clump contains an average of 20–30 culms and there may be >80 culms in very large clumps (An *et al.* 2010). In Yunnan, *D. membranaceus* usually develops into extensive natural bamboo forests along the lower Lancang River watershed over an area of ca. 7 × 10^4^ h m^2^ (Xie *et al.* 2016). *Dendrocalamussinicus* grows 20–35 m in tall and 10–30 cm in diameter and it is regarded as the strongest woody bamboo species in the world (Dong *et al.* 2012). Every clump contains 20–25 culms and there can be >90 culms in large clumps (Hui 2004). Local people practice small-scale cultivation of this species using clones obtained by dividing rhizomes. There was a continuous period of flowering and fruiting amongst different clumps and populations of *D. membranaceus* and *D. sinicus* in Yunnan associated with severe droughts from 2008 to 2015 (Gu *et al.* 2012; Xie *et al.* 2016). In the wild, *D. membranaceus* populations had both sporadic and mass flowering and fruiting, whereas only sporadic flowering and fruiting was seen in *D. sinicus* populations. The present study was conducted from March 2013 to December 2015 and focused on populations of *D. sinicus* and *D. membranaceus* with a similar flowering period.

### Study sites

**Figure 2.**
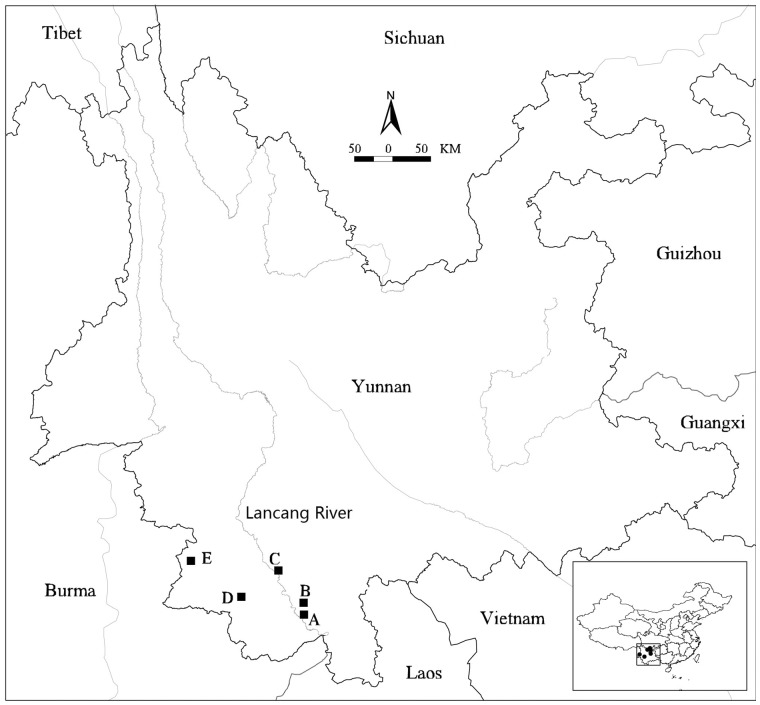
Location of study sites for breeding system and pollination biology of *D. membranaceus* (sites A, B and C) and *D. sinicus* (sites D and E) in Yunnan, China.

Breeding system and pollination surveys of *D. membranaceus* were conducted in the bamboo-dominated forests along the Lancang River, including Xiaomengyang National Nature Reserve (XNNR) in Jinghong County (sites A and B) and Simao District of Puer City (site C; Fig. 2). The XNNR is located at 21°48′–22°20′N, 100°36′–101°22′E, with an elevation range of 600–900 m (Xie *et al.* 2016). The observation sites A (for mass flowering), B and C (for sporadic flowering) were selected for investigating breeding system and pollination in mass and sporadically flowering populations of *D. membranaceus* from the beginning of 2013. Site A was in pure *D. membranaceus* forest in which 52 of 61 clumps flowered (85.2 %), and four flowering clumps were randomly selected for breeding system studies. Site B was in a mixed forest of bamboo and broadleaf trees (bamboo: tree = 7:3) where 4 clumps of 32 clumps flowered (12.5 %), and all four flowering clumps were studied. Site C was also in a mixed bamboo-tree forest (bamboo: tree = 9:1), and 3 of 26 clumps flowered (11.5 %) (Table 1). We observed all three flowering clumps at site C. Field studies of *D. sinicus* were conducted in the cultivated populations in Menghai County of Xishuangbanna Autonomous Prefecture (site D) and Ximeng County of Puer City (site E), where *D. sinicus* was found sporadically flowering (Table 1; Fig. 2). Three and two flowering clumps were studied, respectively.

### Floret morphology and development

We considered each bamboo clump as a probable genet and the culms within as ramets of a clone according to McClure (1966). During the full blooming stage of *D. membranaceus* and *D. sinicus*, 30 intact fresh florets were selected and measured from every clump surveyed. Floret morphological characters were observed using a ×10 hand lens. Glumes, lemmas, paleas, filaments, anthers and styles of the floret were measured for length and median width (max-width) using vernier calipers. To reveal details of floral development, such as the floral development sequence, 10 pseudospikelets from each flowering culm were observed.

### Breeding system

To assess the effects of pollen source on seed set of *D. membranaceus* and *D. sinicus*, various pollination experiments were performed *in situ* during the spring of 2014 and 2015. Because the flowering bamboo clumps will die within one year, we had only one chance to apply the experimental treatments to each flowering bamboo clump. For each clump, at least five flowering culms were selected to study the breeding system. Pollination treatments, performed according to Dafni (1992) and Gan *et al.* (2013) were conducted as follow: natural control, no emasculation and no bagging; autonomous self-pollination, no emasculation and bagging to test whether pollinators were needed; assisted geitonogamy, emasculation and bagging to test whether geitonogamy was limited; assisted xenogamy, emasculation and bagging to test fertility of outcrossing; natural cross-pollination, emasculation and no bagging to test whether pollen transfer was limited; parthenogenesis test, emasculation, bagging and no pollination by hand to test whether agamospermy may have occurred.

Hand pollination experiments were carried out according to Zych and Stpiczyńska (2012). Pollen was collected from non-dehisced ripe anthers of either the same individual flower (self-pollination) or another flower from three unmarked clumps of the same species that was growing at a distance of at least 5 m (cross-pollination). Pollen was stored in 1.5 mL Eppendorf tubes. All pollen was applied within 4 h to ensure viability. When stigmas were visible, pollen was applied to florets using one sterile brush for each species. Three replicates of 20 pseudospikelets were marked for each treatment. For *D. membranaceus*, because only the floret at the top of a pseudospikelet was pollinated and eventually developed into a seed, we used only the topmost floret for pollination studies. In *D. sinicus*, the topmost floret of each pseudospikelet was sterile and most of pseudospikelets only bear one seed from the second floret so we only used the second floret in the hand pollination experiments. Seed set was estimated with the following formula: seed set (%) = total number of seeds/total number of pseudospikelets × 100 %.

The pollen limitation index (*L*; Larson and Barrett 2000) was used to quantify pollen limitation within a population for each species. The index was calculated as *L* = 1 - (*P_N_*/*P_S_*), where *P_N_* is the percentage of naturally pollinated seeds and *P_S_* is the percentage seed set by supplement cross-pollen. No pollen limitation is indicated in the population if *L *= 0 (Larson and Barrett 2000).

### Flower visitors

Flower visitor activities were observed in the field at the peak of flowering in late March of 2014 and 2015, respectively. Observations were conducted simultaneously at three sites for *D. membranaceus* and *D. sinicus* from 0800 h to 1600 h on 7 sunny days. For each observation we selected 10 flowering branches each having at least ten blooming pseudospikelets. Representative flower visitors were captured for identification using trap nets. It was recorded if visitors carried *D. membranaceus* and *D. sinicus* pollen. Pollinator frequency was expressed as number of visits to florets per hour.

### Statistical analysis

Seed set of the hand pollination experiments was compared by a one-way ANOVA using PROC GLM. When significant differences were found, means were separated by Student–Newman–Keuls (SNK) multiple comparison analysis at *P *= 0.01 and *P *= 0.05, respectively. All statistical analyses were performed with SAS (Version 9.2, SAS Institute Inc., Cary, NC).

The statistical significance of pollen limitation was evaluated by Student's *t* test (independent samples) in Excel 2010 (Microsoft Corp., Seattle, WA). Differences were considered to be statistically significant when *P *≤ 0.05 and highly significant when *P *≤ 0.01.

## Results

### Floret morphology

**Figure 3.**
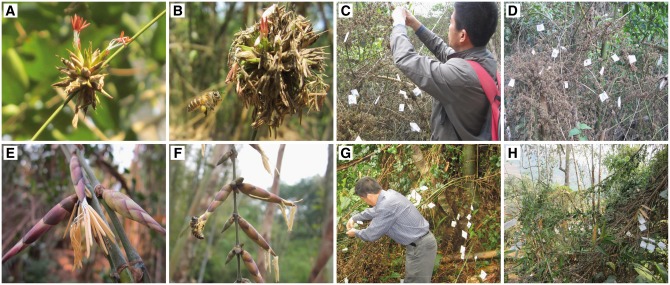
Inflorescences and hand pollination experiments of *D. membranaceus* (A‒D) and *D. sinicus* (E‒H).

Both *D. membranaceus* and *D. sinicus* bear large-scaled panicles on leafless flower branches (Fig. 3), but they differed in floral morphology. In *D. membranaceus*, 30–70 (minimum and maximum) pseudospikelets form a dense, spiky globose mass at the nodes of flowering branches and this was 2.5–3.5 cm in diameter. Pseudospikelets are flat, ovoid, 10–12 mm long, 2–4 mm wide, yellow green when fresh and straw yellow when withered. Each pseudospikelet comprises 2–4 florets. The floret bears two ovate, apically acute glumes. Lemma is 8–9 mm long, 5–8 mm wide. Palea is membranous, 7–8 mm long, 3–6 mm wide, with two ciliate keels. Stamens protruded from a floret when mature. Filaments are milky white, 10–15 mm long. Anthers are purple, 5–8 mm long. Ovary is ovoid and hairy, and ciliate style is 10–15 mm long with one purple and plumose stigma.

In *D. sinicus*, 1–6 pseudospikelets form a sparse cluster at each node of flowering branches. Pseudospikelets are flat, narrowly ovoid, 30–45 mm long, 5–8 mm wide, purple when fresh and also straw yellow when withered. Each pseudospikelet contains 6–7 florets. Floret bears two ovate and apically acute glumes. Lemma is 10–25 mm long and 10–15 mm wide. Palea is 12–18 mm long and 6–12 mm wide, membranous, two-keeled, with a two-fid apex. Six stamens protruded from the floret when it matured. Filaments are milky white, 20–35 mm long. Anthers are 8–15 mm long, yellow, with mucronate apices. Ovary is ovoid and hairy, and ciliate style is 20–35 mm long with only one purple and plumose stigma.

### Floret development

**Figure 4.**
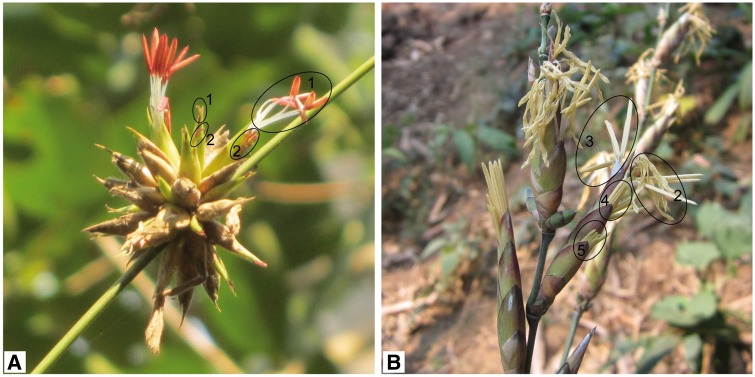
Floret development within a single pseudospikelet of *D. membranaceus* and *D. sinicus*. The number indicates the order of the floret in the pseudospikelet.

March 2013–December 2015 observations revealed that *D. membranaceus* and *D. sinicus* bloom at any time of the day but mostly occurred from 8000 h to 1200 h. Both species were protogynous but the maturation of pistils and stamens of different florets can overlap (Fig. 4). Typically, extrusion of the stigma from the lemma occurs 4 h earlier than the stamens. In *D. sinicus*, the flowering duration of a single pseudospikelet (from protrusion of the stigma of the uppermost fertile floret to withering of the stigma of the last maturing floret) lasts up to 30 h, which was 10 h longer than that of *D. membranaceus.*

Florets in the same pseudospikelet matured at different times. In *D. membranaceus*, the topmost floret matured first, but the basal florets often degenerated. In *D. sinicus*, the topmost floret was vestigial, and florets matured sequentially from the second floret downwards, i.e. in a basipetal manner (Fig. 4).

### Breeding system

**Figure 5.**
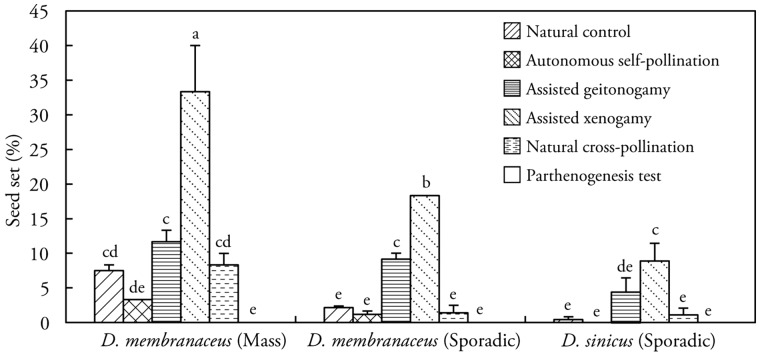
Average seed set of *D*. *membranaceus* and *D. sinicus* from different flowering populations subjected to six pollination treatments. Error bars are 95 % confidence intervals for the mean. Significant differences were detected between treatments in every population studied (*F*_17, 36_ = 60.43, *P *< 0.0001 for both variables). The results of the same treatments were compared across populations, and means with the same letter are not significantly different at *P *< 0.05.

Seeds were produced after the geitonogamy and xenogamy fertilization treatments (Fig. 5), indicating that *D. membranaceus* and *D. sinicus* are self-compatible and outcrossing fertile. However, results from a one-way ANOVA indicated that a highly significant difference of seed set (*F*_17,__ __36_ = 60.43, *P *< 0.0001) existed between the assisted geitonogamy and assisted xenogamy in five study sites (sites A–E). These sites included mass flowering populations and sporadically flowering populations of *D. membranaceus* as well as sporadically flowering populations of *D. sinicus*. This suggests that these two species are pre-dominantly outcrossing (*P *< 0.01). Seed set from assisted geitonogamy was significantly higher (*P *< 0.05) than seed set from autonomous self-pollination, and there was no significant difference between natural pollination and autonomous self-pollination and natural cross-pollination (Fig. 5), suggesting pollination limitation in those populations. No seeds were obtained with emasculation and bagging, suggesting absence of agamospermy in two bamboo species.

Seed set in *D. sinicus* was 0.42 ± 0.42 % under natural pollination in sites D and E. After artificial xenogamy, seed set increased 20-fold to 8.89 ± 2.55 %. Seed set of *D. membranaceus* was relatively higher. Results from a second ANOVA revealed that there was a significant difference between mass flowering (7.49 ± 0.82 %) and sporadically flowering (2.14 ± 0.25 %) populations with natural pollination (*F*_11__, __24_ = 63.86, *P *< 0.0001; Fig. 5). There was also a significant difference in seed set from assisted xenogamy and natural cross-pollination (*P *< 0.01) between flowering populations of *D. membranaceus* (*F*_11__, __24_ = 63.86, *P *< 0.0001; Fig. 5), but no significant differences in seed set from autonomous self-pollination and assisted geitonogamy.

### Pollination limitation

Pollen limitation (based on comparison of natural pollination with supplemental pollination) occurred in sporadically flowering natural populations of *D. membranaceus* as well as in the cultivated stand of *D. sinicus*. The pollen limitation index (*L*) values were 0.895 and 0.961, respectively (Table 2), and the difference was highly significant (*P *< 0.01) in *D. sinicus*, which had higher mean seed set with supplemental pollination (10.67 ± 0.82) compared with open pollination (0.42 ± 0.42). In the mass flowering *D. membranaceus* population there was no significant pollen limitation although seed set under natural population (7.49 ± 0.82) was significantly increased seed set (31.17 ± 0.63) in the supplemented treatment.

**Table 1.** Main characteristics of the study sites for *D. membranaceus* (sites A, B and C) and *D. sinicus* (sites D and E). Due to scarcity of flowering bamboo clumps, we selected a total of 10 flowering clumps for a breeding system study in sites A (four clumps), B (four clumps) and D (two clumps) in March 2014, and six flowering clumps in sites C (three clumps), D (one clump) and E (two clumps) in March 2015.

Species	Study site	Longitude (E)	Latitude (N)	Elevation (m)	Type and number of flowering bamboo groves
*D. membranaceus*	A: pure forest	100°52′28′′	22°01′52′′	753	Mass flowering, 52 of 61 clumps flowered, and four flowering clumps were randomly selected for breeding system studies.
	B: bamboo-tree mixed forest (bamboo: tree = 7:3)	100°52′08′′	22°10′28′′	831	Sporadic flowering, 4 clumps of 32 clumps flowered, and all four flowering clumps were studied.
	C: bamboo-tree mixed forest (bamboo: tree = 9:1)	100°33′35′′	22°34′01′′	751	Sporadic flowering, 3 of 26 clumps flowered, and all three flowering clumps were studied.
*D. sinicus*	D: cultivated stand	100°05′98′′	22°14′50′′	925	Sporadic flowering, three flowering clumps were studied.
	E: cultivated stand	99°29′32′′	22°41′18′′	975	Sporadic flowering, two flowering clumps, both were studied.

**Table 2.** Pollen limitation value in *D. membranaceus* and *D. sinicus*. Twenty pseudospikelets were randomly selected in each group. In *D. sinicus*, two florets at the top of the pseudospikelet were observed because the topmost floret was often sterile. In *D. membranaceus*, only the first floret at the top of the pseudospikelet was observed. Asterisks indicate that the pollination limitation was significant at the *P *< 0.05 (*) and *P *< 0.01 (**) levels.

Species	Flowering type	Seed set in the natural pollination (%)	Seed set in the supplemental cross-pollen (%)	Pollen limitation index (*L*)	Significance test (*t* test)
*D*. *membranaceus*	Mass	7.49 ± 0.82	31.17 ± 0.63	0.760	–
*D*. *membranaceus*	Sporadic	2.14 ± 0.25	20.39 ± 1.00	0.895	*
*D. sinicus*	Sporadic	0.42 ± 0.42	10.67 ± 0.82	0.961	**

### Flower visitors

**Figure 6.**
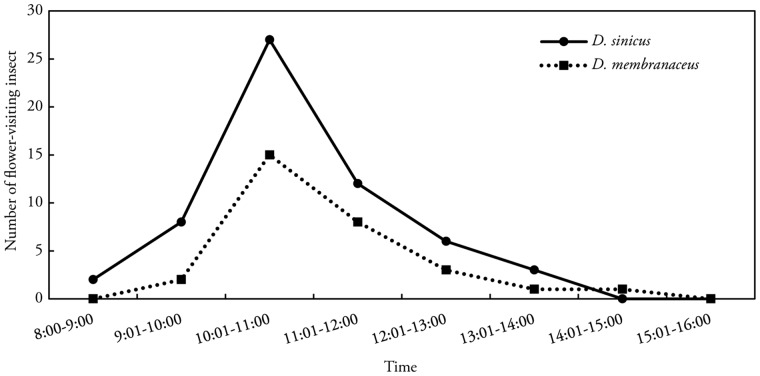
Flower visitation of the Asian honeybee to flowering *D. membranaceus* and *D. sinicus* bamboos on sunny days.

Although both *D. membranaceus* and *D. sinicus* were expected to be wind pollinated, insect flower visitors were observed at five study sites. We observed a total of 48 and 82 insect visits to *D. membranaceus* and *D. sinicus* flowers, respectively. The Asian honeybee *Apis cerana* was the most common insect visitor (Fig. 6). On sunny days, the mean visitation frequency of *A. cerana* was 11.5 and 19.5 times per hour for *D. membranaceus* and *D. sinicus*, respectively. Visitation frequency peaked from 1000 h to 1200 h for both bamboo species. Thereafter, the number of visiting bees rapidly declined. Cloudy or rainy days also reduced visitation frequency (Fig. 6).

Honeybees were often observed visiting more than one flower per clump, and generally had short flight ranges. Most visitors grasped anthers and gathered pollen grains with their forelegs, then transferred grains to their hindlegs and abdomen. At the same time, masses of grains were dispersed into the air, accentuated when more bees visited flowers.

## Discussion

### Breeding system

*Dendrocalamus* *membranaceus* and *D. sinicus* are self-compatible but pre-dominantly outcrossing, and no apomixis occurs. The results are consistent with the findings of Venkatesh (1984) and Kitamura and Takayuki (2011) who reported that bamboo species such as *Bambusa arundinacea* and *Sasa cernua*, were self-compatible because seed set was observed even with imposed (bagged) isolation and without emasculation. In this study, the seed set from xenogamy and the control was greater than those from autogamy, suggesting that outcrossing is pre-dominant in the breeding system of *D. membranaceus* and *D. sinicus* and similar to other bamboos such as those reported by Gan *et al.* (2013). Higher seed set in the two species from assisted geitonogamy compared with autonomous self-pollination implied that self-fertilization may depend on pollen vectors. The seed set from autonomous self-pollination and assisted geitonogamy were similar in mass and sporadic flowering populations of *D. membranaceus*, suggesting stable self-fertility. But in *D. sinicus*, the self-compatibility may be dependent on pollen vectors such as honeybees because bagging experiments confirmed the absence of auto-fertility. These results are similar with those of Johnson *et al.* (2006) and Blambert *et al.* (2016). In natural populations of *D. membranaceus* and *D. sinicus*, the higher seed set rates in assisted xenogamy treatments reflected a strong tendency for xenogamy and indicated either a lack of pollinators or low pollination efficiency. Self-compatibility may be the evolutionary consequence of lack of pollinators or inadequate pollination (Barrett 1998; Jacquemyn *et al.* 2005; Blambert *et al.* 2016).

Mating systems influence the ecology and evolutionary dynamics of plant populations (Kittelson and Maron 2000; Nair *et al.* 2004; Mousset *et al.* 2016). Many plant species employ a mixed mating strategy (Lord 2015), and this has been reported for the Poaceae such as *S.cernua* (Kitamura and Takayuki 2011) and *Sorghum bicolor* (Muraya *et al.* 2011). *Dendrocalamusmembranaceus* and *D. sinicus* also demonstrate a mixed mating system with pre-dominant outcrossing. They are both self-compatible but pollinator-dependent in *D. sinicus* and less so in *D. membranaceus*.

### Pollination

Pollination of seed plants is an essential step in their sexual reproduction (Memmott *et al.* 2007). *Dendrocalamusmembranaceus* and *D. sinicus* are wind pollinated, as in most woody bamboos such as *Phyllostachys pubescens* (Cheung *et al.* 1985), *Dendrocalamus strictus* (Nadgauda *et al.* 1993) and *Arundinaria gigantea* (Gagnon and Platt 2008). However, pollinators such as *Apis mellifera*, have been observed visiting the flowers of some bamboos (Venkatesh 1984; Nadgauda *et al.* 1993). Their role in the pollination of bamboos was overlooked because they only seemed to gather pollen in the male phase of flowering and neglected the female phase. As such, they were only considered to be vectors of pollen (Nadgauda *et al.* 1993). In our study, honeybees at a site visited flowers sequentially and carried pollen away on their hindlegs, increasing the likelihood of transferring pollen from flowers of the same or nearby flowering bamboo clumps. Honeybees may be good pollinators for *D. membranaceus* and *D. sinicus*. These two bamboo species may have evolved to release pollen that is available for bee pollination. The time of pollen release may coincide with the peak of their flower visitation activity. Masses of pollen grains were released into the air when bees visited flowers and this would increase the chances for geitonogamy pollination. Our observations suggest that honeybee pollination could augment wind-pollination in *D. membranaceus* and *D. sinicus*, similar to study on *Phyllostachys nidularia* by Huang *et al.* (2002).

### Natural vs. artificial population variation in breeding system

Habitat fragmentation and destruction affect plant reproduction by reducing the availability, and increasing the distance between, potential mates (Larson and Barrett 2000; Blambert *et al.* 2016). In the present study, the seed set of *D. sinicus* was significantly lower than that of the mass-flowering population of *D. membranaceus*, but was similar to that of the sporadically flowering populations of the latter. Pollen limitation may have contributed to the low seed set but this could be overcome by assisted pollination, and the increase was more evident in *D. sinicus*. Generally, pollen limitation results from a reduction in the quantity and/or quality of pollen deposited on stigmas, leading to lower ovule fertilization and seed production (González-Varo *et al.* 2009; Agulló *et al.* 2015). In the present study, the spatial distribution of individuals in a population and pollinator presence and activity, may have helped to reduce pollen limitation. These factors also influenced pollination in *Phyllodoce aleutica* (Kameyama and Kudo 2009). The spatial separation of flowering clumps could reduce opportunities for outcrossing in both the sporadic flowering cultivated stands of *D. sinicus* and the natural populations of *D. membranaceus*. Inbreeding, through self-fertilization, may cause an increase in homozygosity amongst offspring, leading to accumulation of deleterious alleles and inbreeding depression (Blambert *et al.* 2016; Xie *et al.* 2016). Such inbreeding depression can be expressed as postzygotically by reduced seed germination in bamboo (Kitamura and Takayuki 2011). Yang *et al.* (2013) found that seeds of *D. membranaceus* had a higher germination rate (81.0 %) than those of *D. sinicus* (43.3 %), though it was unclear if inbreeding depression was involved.

### Conservation biology

Given the rapid decline of *D. membranaceus* and *D. sinicus* populations (Gu *et al.* 2012; Xie *et al.* 2016), immediate conservation efforts should be considered to protect their germplasm. The genetic diversity of the species would be at risk of further decline, so both *in situ* and *ex situ* conservation measures may be necessary for these species. For *D. membranaceus*, management of the species could involve enlarging or retaining populations with genetically less-related individuals, i.e. ‘genetic rescue’ (Holmes *et al.* 2008). Meanwhile, closing the land for reforestation and prohibiting human intrusion would help natural recovery processes in the mass-flowering populations (Xie *et al.* 2016). Attention should be directed toward the conservation of *D. sinicus* due to its limited distribution and unsupervised collection of culms (Hui 2004). One practical conservation solution would be to establish more *ex situ* cultivated stands to meet commercial demands. We should also make a special effort to collect seeds of *D. membranaceus* and *D. sinicus* for study. More research is needed to determine whether inbreeding depression is occurring in their flowering populations, especially in the sporadic flowering populations.

## Conclusions

This is the first report on variation in breeding system from mass and sporadically flowering bamboo populations. Our results demonstrate that both *Dendrocalamus* species have a mixed mating system with self-compatibility, pre-dominant outcrossing and no agamospermy. The selfing component may be dependent on a pollen vector for seed setting, particularly in *D. sinicus*. Reproductive limitations were revealed in the sporadically flowering populations of *D. sinicus*. Pollen limitation and scarcity of wild pollinators influenced the sexual reproduction of *D. membranaceus* and *D. sinicus*. Honeybees may effectively augment wind pollination in sporadically flowering populations of both species.

## Sources of Funding

The research was supported by the Fundamental Research Funds of the Chinese Academy of Forestry (CAFYBB2017ZX001-8), the National Natural Science Foundation of China (31270662), Department of Sciences and Technology of Yunnan Province (2014HB041, 2008OC001).

## Contribution by the Authors

L.-N.C., Y.-Z.C. and H.-Q.Y. conducted experiment work, analysed the data; L.-N.C., Y.-Z.C., K.-M.W. and H.-Q.Y. wrote the paper; H.-Q.Y. and D.-Z.L. conceived the experiments.

## Conflicts of Interest Statement

None declared.
